# The Interdomain Linker of AAV-2 Rep68 Is an Integral Part of Its Oligomerization Domain: Role of a Conserved SF3 Helicase Residue in Oligomerization

**DOI:** 10.1371/journal.ppat.1002764

**Published:** 2012-06-14

**Authors:** Francisco Zarate-Perez, Martino Bardelli, John W. Burgner, Maria Villamil-Jarauta, Kanni Das, Demet Kekilli, Jorge Mansilla-Soto, R. Michael Linden, Carlos R. Escalante

**Affiliations:** 1 Department of Physiology and Biophysics, Virginia Commonwealth University, School of Medicine, Richmond, Virginia, United States of America; 2 Department of Infectious Diseases, King's College London School of Medicine at Guy's, King's and St. Thomas Hospital, London, United Kingdom; 3 Department of Applied Sciences, University of the West of England, Bristol, United Kingdom; 4 Center for Cell Engineering, Department of Human Genetics, Memorial Sloan-Kettering Cancer Center, New York, New York, United States of America; 5 UCL Gene Therapy Consortium, UCL Cancer Institute, University College London, London, United Kingdom; Penn State University School of Medicine, United States of America

## Abstract

The four Rep proteins of adeno-associated virus (AAV) orchestrate all aspects of its viral life cycle, including transcription regulation, DNA replication, virus assembly, and site-specific integration of the viral genome into the human chromosome 19. All Rep proteins share a central SF3 superfamily helicase domain. In other SF3 members this domain is sufficient to induce oligomerization. However, the helicase domain in AAV Rep proteins (i.e. Rep40/Rep52) as shown by its monomeric characteristic, is not able to mediate stable oligomerization. This observation led us to hypothesize the existence of an as yet undefined structural determinant that regulates Rep oligomerization. In this document, we described a detailed structural comparison between the helicase domains of AAV-2 Rep proteins and those of the other SF3 members. This analysis shows a major structural difference residing in the small oligomerization sub-domain (OD) of Rep helicase domain. In addition, secondary structure prediction of the linker connecting the helicase domain to the origin-binding domain (OBD) indicates the potential to form α-helices. We demonstrate that mutant Rep40 constructs containing different lengths of the linker are able to form dimers, and in the presence of ATP/ADP, larger oligomers. We further identified an aromatic linker residue (Y224) that is critical for oligomerization, establishing it as a conserved signature motif in SF3 helicases. Mutation of this residue critically affects oligomerization as well as completely abolishes the ability to produce infectious virus. Taken together, our data support a model where the linker residues preceding the helicase domain fold into an α-helix that becomes an integral part of the helicase domain and is critical for the oligomerization and function of Rep68/78 proteins through cooperative interaction with the OBD and helicase domains.

## Author Summary

Viruses have to optimize the limited size of their genomes in order to generate the proteins required for infection and replication. Several mechanisms are used to accomplish this including the use of multiple promoters and alternative splicing. These processes generate gene products with diverse functions through the combinatorial assembly of a small number of protein domains. The small genome of the adeno-associated virus has two major open reading frames that generate seven proteins, four non-structural Rep proteins and three capsid proteins. The non-structural Rep proteins share a motor domain that uses hydrolysis of ATP to generate the conformational changes that drive DNA replication, transcriptional regulation, site-specific integration and the packing of viral genome into capsids. These functions depend upon the oligomerization of Rep proteins on specific DNA sites through the cooperation of the N-terminal origin binding domain and the C-terminal helicase domain. We provide evidence that the linker that connects the two domains is an integral feature of the helicase domain and contains a conserved aromatic residue that is critical for oligomerization. This residue emerges to be a signature motif of SF3 helicases and is also present in a subset of bacterial Rep proteins that support rolling circle replication mechanism.

## Introduction

The four adeno-associated virus (AAV) Rep proteins are generated from a single open reading frame by the transcriptional use of two different promoters (p5 and p19) and subsequent alternative splicing mechanisms [1], [2], [3]. These reactions produce proteins that share three functional domains: an origin binding domain (OBD), a SF3 helicase domain and a putative zinc-finger domain [4], [5]. The combination of these domains imparts these proteins with striking multifunctionality. In particular, the larger proteins Rep78 and Rep68 function as initiators of DNA replication, transcriptional regulators, DNA helicases and as key factors in site-specific integration [6]. The smaller Rep proteins Rep40 and Rep52, play a critical role during packaging of viral DNA into preformed empty capsids, where they are thought to be part of the packaging motor complex [7], [8], [9]. Although in terms of domain architecture the AAV Rep proteins resemble other members of the SF3 protein family, the peculiar OBD with its additional nuclease activity and the complex character of their oligomeric properties, set them apart from other SF3 helicases such as simian virus 40 large T antigen (SV40-LTag) and papilloma virus E1 (PV-E1) proteins [10], [11], [12], [13]. In both of these proteins, the minimal SF3 helicase domain assembles into a hexameric ring in a process that can be induced by the presence of ATP and/or single-stranded DNA [14], [15]. In contrast, Rep40 containing only the helicase domain and Rep52 with an additional Zn-finger domain, appear to be monomeric [16], [17]. This indicates that oligomerization of AAV Rep proteins requires the presence of both the OBD domain and the helicase domain. This combination imparts both Rep68 and Rep78 with a complex and dynamic oligomeric behavior *in-vitro* that is modulated in large part by the nature of the DNA substrate [18]. The monomeric behavior of both Rep40 and Rep52 is striking in that they appear to contain the required structural features that are present in other SF3 helicase members. The X-ray structures of both SV40-LTag and PV-E1 show that their helicase domains assemble as hexameric rings and that the oligomerization interface is bipartite [15], [19]. One interface is formed by the interaction of neighbouring N-terminal oligomerization domains (OD). The second interface is formed by the interaction of the C-terminal AAA^+^ domains and is further stabilized by the presence of nucleotides [11], [15]. In order to understand the structural features that promote AAV Rep oligomerization, we pursued in this study a detailed structural comparison of SF3 helicases. We show that the OD domain in Rep40/52 has been hindered in its ability to oligomerize by the transcriptional use of the p19 promoter. This event generates proteins with a smaller OD domain as compared to other SF3 helicases. More importantly, we show that in the context of Rep68/78 the required oligomerization is supported by the interdomain linker which is directly involved in oligomerization interface and we provide evidence that the tyrosine residue preceding the start of Rep40/52 (Y224) is critical in the oligomerization and therefore activity of the large AAV Rep proteins. Taken together, our results support a model where oligomerization of Rep68/78 is mediated by a composite oligomerization interface formed by the OBD, helicase and linker domains, with the latter playing an essential role in the inducing the oligomerization process.

## Results

### The oligomerization domain (OD) of AAV Rep40 differs from the OD's of other hexameric SF3 helicases

**Figure 1:**
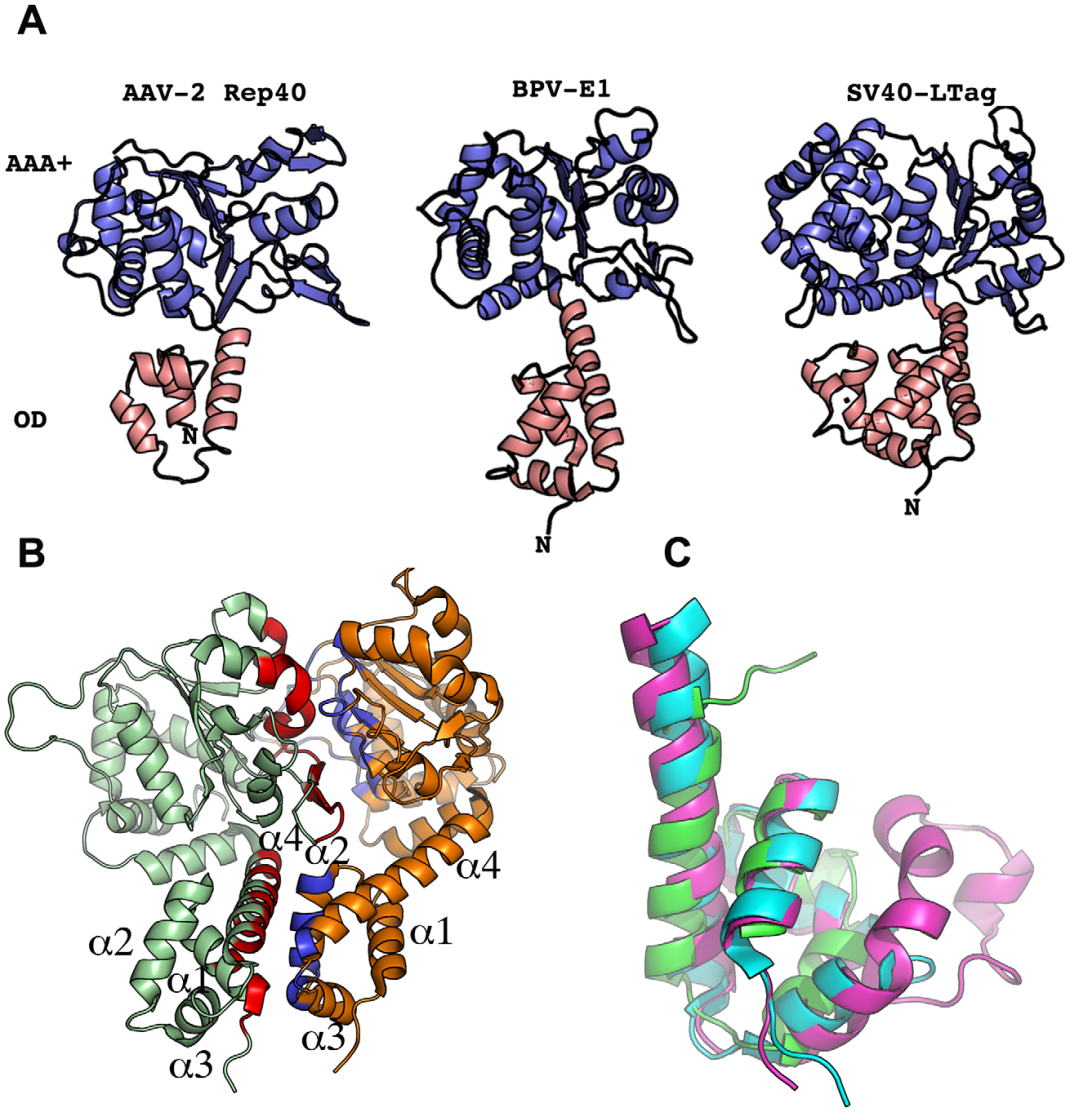
Structural comparison of SF3 helicase structures. (A) Ribbon representation of SF3 helicases AAV-2 Rep40, PV-E1 and SV40-LTag. Salmon color depicts the oligomerization domain (OD). Blue color represents the AAA^+^ domain. (B) PV-E1 dimer showing the residues participating in the formation of the oligomerization interface colored in red and blue. (C) Structural alignment of the OD domain of AAV-2 Rep40 (Green), BPV-E1(Blue) and SV40-LTag(Magenta).

As a first step in our attempt to determine the structural features that promote oligomerization in AAV Rep proteins, we analyzed the oligomeric interface of SF3 family members SV40-LTag and PV-E1. As previously described, the helicase domain contains two subdomains: a N-terminal helical bundle of four α-helices known as the oligomerization domain (OD) and the C-terminal AAA^+^ subdomain (Figure 1A). In PV-E1 the oligomerization interface spans both subdomains forming two extended surfaces at opposite faces of the proteins. In the AAA^+^ subdomain, one face comprises all the catalytic residues, including: the P-loop, its subsequent helix, the β-strands with the associated Walker B residues, sensor 1 motif, and one side of the β-hairpin (Figure 1B). The neighboring subunit interacts through areas that are located in the α-helices “behind” the β-sheet and on the opposite side of the β-hairpin (Figure 1B). Overall, about 20% of the solvent accessible area takes part in the interface and includes about 34% of all residues. In PV-E1, the OD domain consists of 68 residues forming a four helical bundle. The oligomeric interface comes from interaction of residues located in helices 1 and 4 in one monomer, with residues in helices 2, 3 and part of helix 4 in the other subunit (Figure 1B). Most of the interface is hydrophobic with many tyrosine and isoleucine residues. Similar types of interactions are seen in the interface formed by the SV40-LTag OD domains. This domain is a lot bulkier, spanning 89 residues that form a five-helix bundle. The extra helix originates from an additional Zn-finger motif. Significantly, the OD of Rep40, on the other hand, has only 52 aminoacids and, thus, is significantly shorter than PV-E1 and SV40-LTag OD domains. The direct result of this difference is a decrease in the total accessible surface area by more than 1000 Å^2^. In addition, the packing of the helices is less compact, producing a more dynamic structure (Figure 1C). We hypothesize that the smaller OD domain of AAV Rep proteins imparts these proteins unique oligomeric properties where the smaller Rep40/52 are mostly monomeric while Rep68/78 -with the additional OBD domain- form oligomers. However, the measurable ATPase activity in all Rep proteins, suggest that Rep40/52 should oligomerize in the presence of nucleotides [20].

### AAV-2 Rep40 forms a transient dimer in the presence of nucleotides

**Figure 2:**
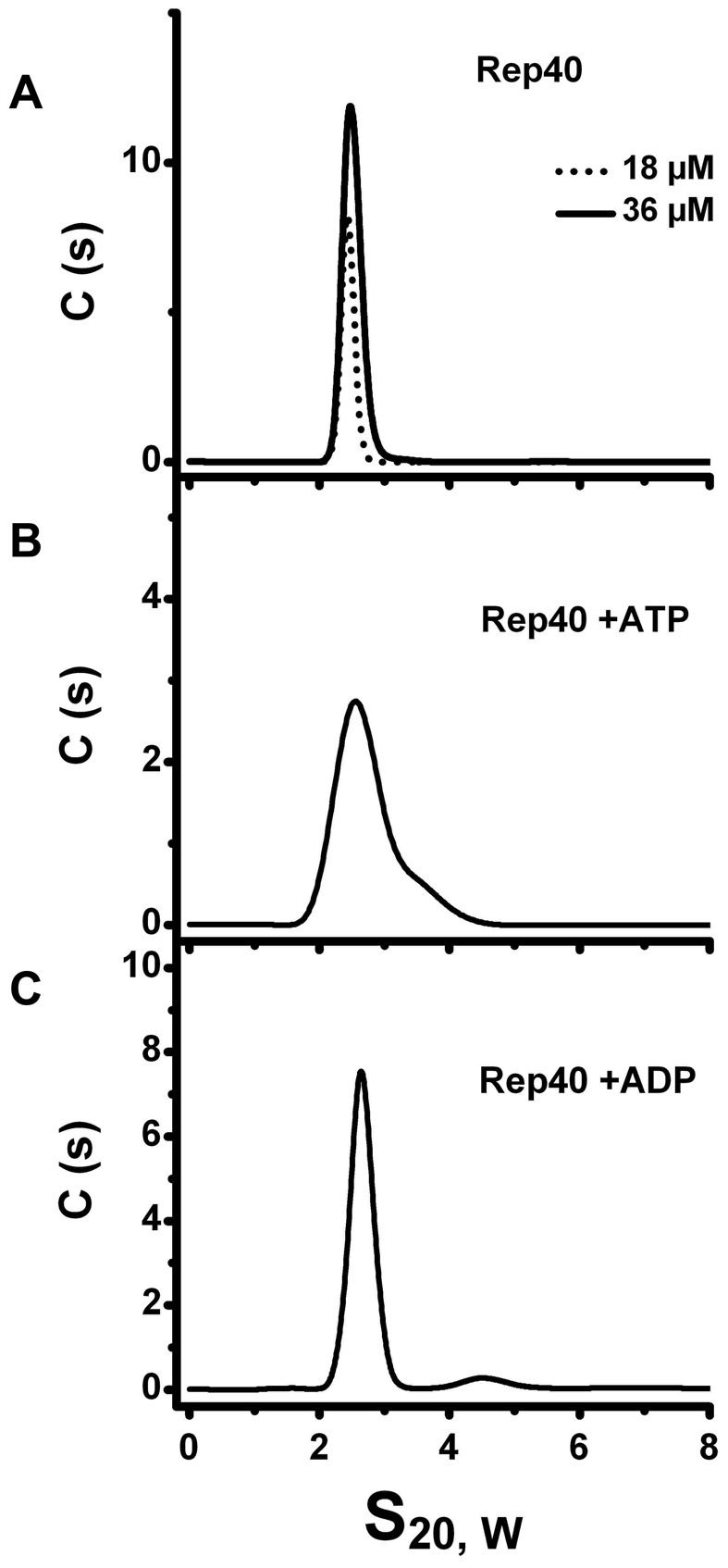
Sedimentation velocity profiles of Re40. (A) Sedimentation velocity analysis of Rep40 at different concentrations (18 µM and 36 µM). In all cases the protein sediments with a sedimentation coefficient of 2.63S. (B) Sedimentation profiles of Rep40 (36 µM) in presence of 5 mM ATP and (C) 5 mM ADP. All the experiments were performed at 40 000 rpm and 20°C in a buffer containing 200 mM NaCl.

To determine if the presence of nucleotides can induce oligomerization of Rep40 -containing the minimal helicase domain-, we carried out sedimentation velocity experiments in the presence and absence of nucleotides at different concentrations. The sedimentation velocity profiles offer a complete characterization of the number and type of oligomers in solution. The data were analyzed using the program sedfit [21], [22]. Figure 2A shows plots of the c(s) distribution against the sedimentation coefficient (s) for two concentrations of Rep40 in the absence of nucleotides. A single peak whose s_20,w_ increases slightly with increasing concentrations is observed. The slight but significant increase in s and calculated molar mass is consistent with a weak and transient dimerization (for hydrodynamic reasons, s is expected to decrease with increasing concentrations of an ideal solute). The data where also fitted using the program sedphat to a monomer-dimer association were the process is in rapid exchange on the time scale of the centrifuge [22]. Table 1 shows that the dissociation constant in the absence of nucleotides is ∼10^−3^ M, which is at the upper end of detection by sedimentation velocity. Similar distributions of Rep40 (at 36 µM) in the presence of either 5 mM ATP or ADP are shown in Figure 2B and 2C. Here an increase is observed in the width of these peaks if compared to those for Rep40 alone. This is a well-understood behavior for a associating system whose exchange kinetics are neither slow of fast on the time scale of the centrifuge, thus, broadening the c(s) distribution peak [23]. The presence of a small shoulder suggest that dimer formation is occurring here as well, although perhaps its rate of dissociation is slower than for Rep40 alone. The s-value of the shoulder is consistent with a transient Rep40 dimer that represents ∼0.2% of the total amount of protein. The relatively low ATPase activity of Rep40 reported in the literature supports our model of transient dimerization promoted by the binding and/or hydrolysis of ATP [20].

**Table 1:** Global fits of the effect of concentration from sedimentation velocity studies on Rep40 and Rep68ΔN200.

Sample	s_monomer_	s_dimer_	logK_a_	K_d_	Global *X^2^*
	S	S		µM	
Rep40	2.63	3.70	3.1	730	0.886
Rep68ΔN200	2.62	4.71	4.1	79	0.20

Sedimentation velocity was performed as described in Materials and Methods. Concentrations of Rep40 used were 18, 36, and 54 µM and for Rep68ΔN200: 4, 8, 16 µM.

### Addition of linker region to Rep40 constructs induces oligomerization

**Figure 3:**
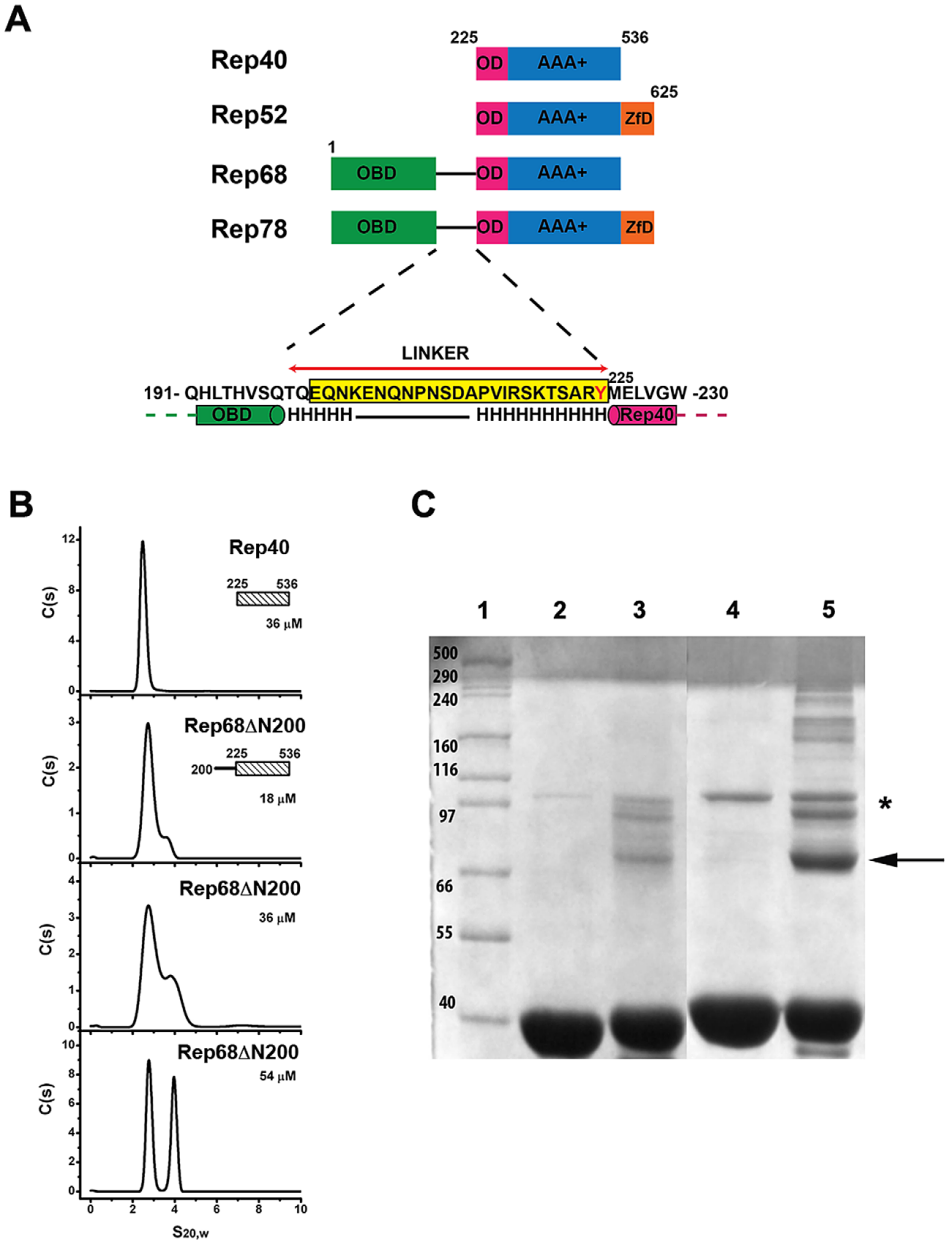
Effect of interdomain linker on oligomerization. (A) A schematic diagram of the AAV-2 Rep proteins with the N-terminal origin binding domain (green), oligomerization domain (pink), AAA^+^ domain (blue) and Zinc finger domain (orange). The sequence of the linker region is shown below with the predicted helical regions connecting the OBD and the helicase domains. Linker sequence is highlighted in yellow. (B) Sedimentation profiles of Rep68ΔN200 over the concentration range from 18 to 54 µM and their comparison with Rep40. The sedimentation analysis shows the presence of two peaks of 2.53S and 3.71S, which corresponds to the monomeric and dimeric species respectively. (C) Cross-linking of Rep40 and Rep68ΔN200. Lane 1, Molecular weight markers; Lane 2, Rep40; Lane3, Cross-linked Rep40; Lane 4, Rep68ΔN200; Lane 5, cross-linked Rep68ΔN200. The arrow shows the position of the Rep68ΔN200 dimer. The asterisk shows proteins bands from non-specific aggregates.

In order to assess whether the interdomain linker connecting the OBD domain and the helicase domains contains additional regions of distinct structure that may play a role in promoting oligomerization, we first carried out secondary structure prediction analysis to determined if the linker contains additional regions of structure. The results suggest that the region from residue 215 to 224 has the potential to form an α-helix (Figure 3A). We hypothesized that this region could extend the first helix of the OD domain (Figure 3A) and the ensuing increase in surface accessible area may be sufficient to drive oligomerization. To test this hypothesis, we designed a new Rep construct beginning at the start of the linker region and extending to aminoacid 536 (a truncated version of Rep68 without the OBD domain, Rep68Δ200), and performed sedimentation velocity and cross-linking studies in order to characterize its oligomerization properties. The sedimentation profile of Rep68ΔN200 shows the presence of two peaks, one corresponding to the monomeric species (∼2.53S) and the other to a dimer (∼3.71S). The amount of formed dimer increases at higher concentrations as expected from a monomer-dimer equilibrium system (Figure 3B). Formation of dimers was also observed when we performed cross-linking experiments. Figure 3C shows that the amount of dimeric species has significantly increased in Rep68ΔN200 as compared to Rep40wt. We calculated the dimerization constants of Rep40wt and Rep68ΔN200 from a global fitting of the sedimentation velocity data to a monomer-dimer model (Table 1). In summary, we determined that the presence of the linker region increases the strength of dimerization by about 10-fold relative to that of Rep40.

### Extension of the linker region to residue 215 defines the minimal length required to promote oligomerization

**Figure 4:**
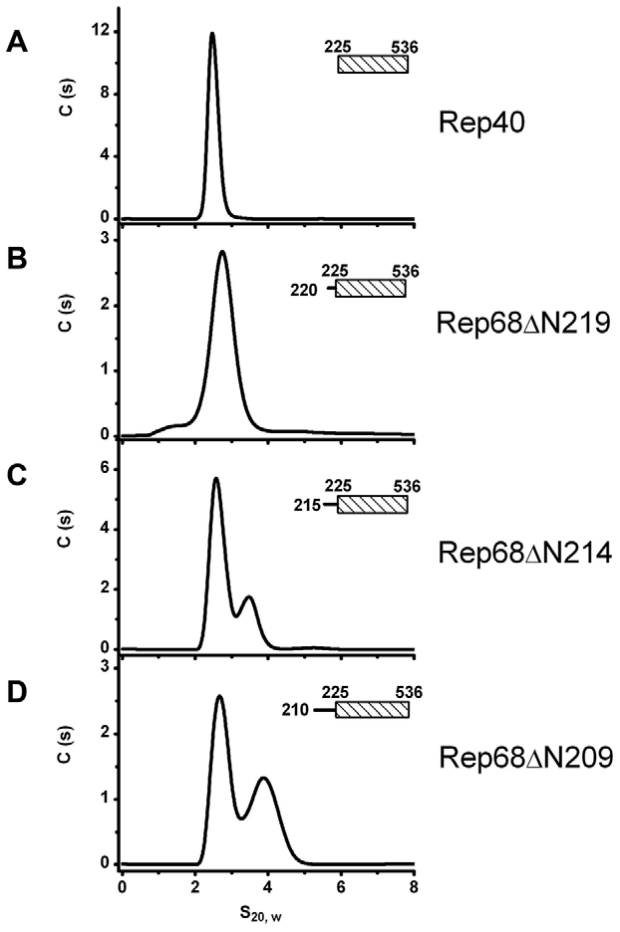
Minimum linker length requirements to induce oligomerization. We generated protein constructs with decreasing lengths of linker that are named in the context of the full Rep68 protein: comparison of the sedimentation velocity profiles of (A) Rep40, (B) Rep68ΔN219 (residues 220–536), (C) Rep68ΔN214 (residues 215–536) and (D) Rep68ΔN209 (residues 210–536). Monomeric species proteins sediment at ∼2.7S while the peak at ∼3.7S corresponds to a dimer. Protein concentration was at 36 µM in all the sedimentation experiments and run at 40000 rpm and 20°C.

Next, we sought to determine the minimal length of linker that is needed to promote oligomerization. We generated three additional constructs, named Rep68ΔN209, Rep68ΔN214 and Rep68ΔN219 and tested their ability to oligomerize (Figure 4). Our results indicate that Rep68ΔN214 contains the minimal length of linker that is required to promote detectible oligomerization, although with the shorter construct Rep68ΔN219, a small shoulder is seen at higher concentration (data not shown). These results confirm that the linker region from 215 to 224 may fold into a α-helix, resulting in an increase of the surface accessible area of the OD domain that mediates oligomerization. This increase, however, is not sufficient to produce higher order oligomers.

### ATP and ADP induce formation of higher order oligomers of the extended linker Rep protein constructs

**Figure 5:**
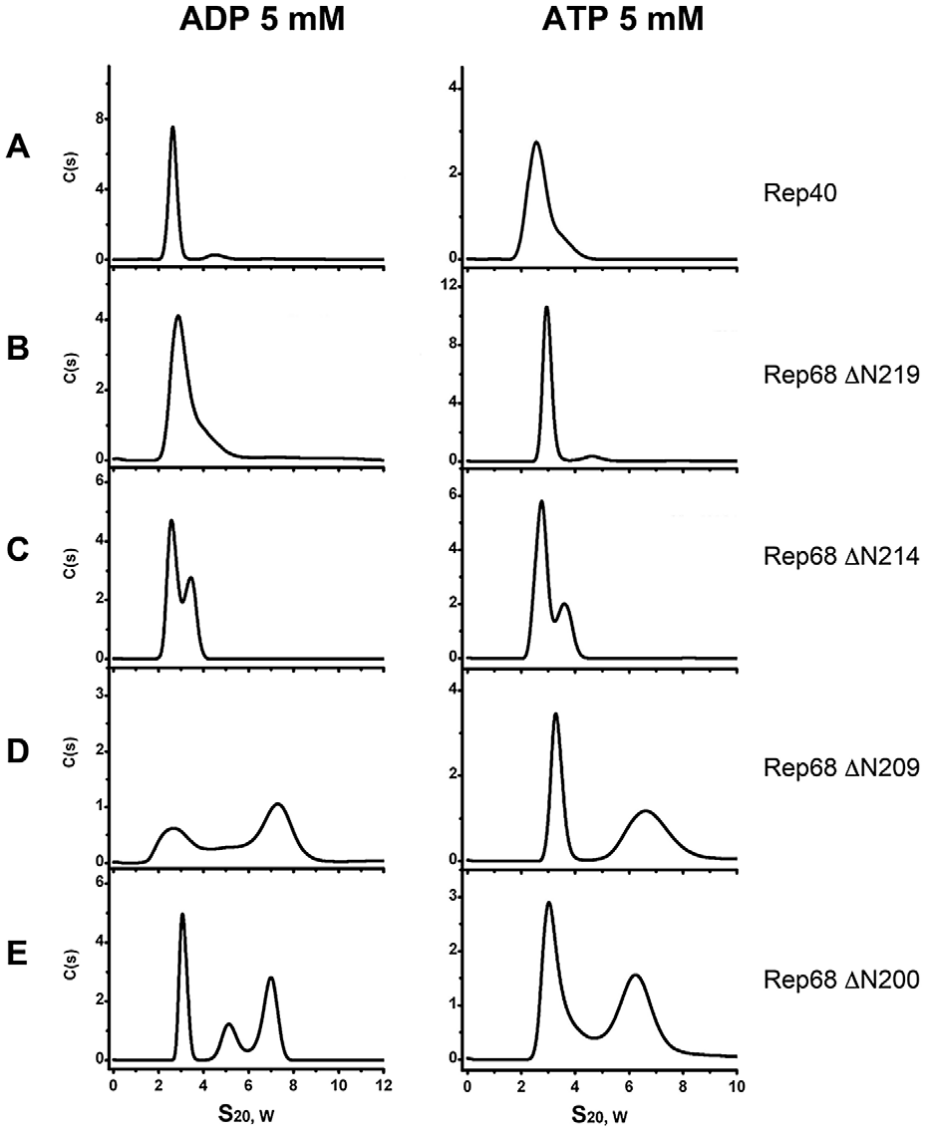
Effect of nucleotides in the oligomerization of Rep extended-linker construct proteins. ATP and ADP were added to each linker construct and compared to Rep40. Sedimentation profiles of (A) Rep40, (B) Rep68Δ219, (C) Rep68Δ214, (D) Rep68Δ209 and (E) Rep68Δ200. All protein concentrations were kept at 36 µM and contain 5 mM of ADP (left panel) or 5 mM ATP (right panel). Sedimentation velocity experiments were run at 40000 rpm and 20°C. Data was collected using the interference system.

In order to determine the contribution of ATP and ADP to the oligomerization of the extended linker Rep linker constructs, we performed sedimentation velocity studies in the presence of nucleotides. Our hypothesis was that if oligomerization reflects the functional state of these proteins, the addition of nucleotides should support and induce further oligomerization. Figure 5 shows that the presence of ATP and ADP induces the formation of higher order oligomers. Formation of dimeric species at this concentration can be seen with Rep68Δ214 as well as the longer constructs RepΔN209 and RepΔN200. In the later two, ADP produces two main populations sedimenting at ∼3S and ∼7S with additional intermediate oligomers. ATP on the other hand, seems to generate more stable species at ∼7S. Again, these data show that the presence of the linker region induces oligomerization of the Rep constructs and that the addition of nucleotides, in particular ATP, induces formation of larger oligomers, possibly through the stabilization of the interface formed by the AAA^+^ domains. This finding is in good agreement with the unique characteristics of the AAV Rep nucleotide binding pocket, which, based on its open conformation together with the presence of an arginine finger predicts the nucleotide contribution to oligomerization [24].

### Linker substitution abolishes oligomerization of Rep68

**Figure 6:**
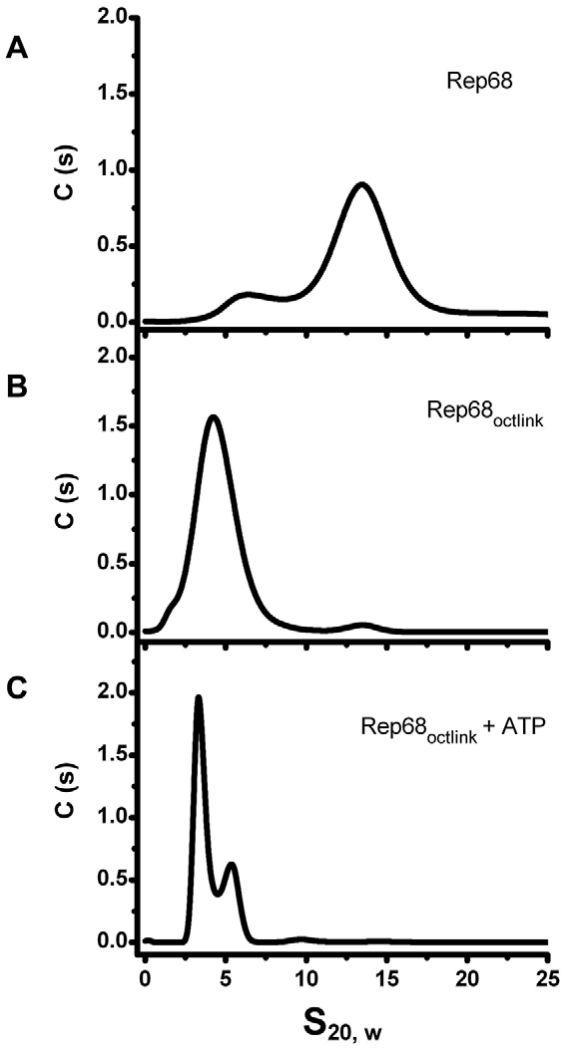
Effect of Linker replacement in Rep68 oligomerization. Comparison of sedimentation profiles of Rep68 and Rep68_octlink_ proteins. (A) Rep68 sediments as a major peak at ∼13S. (B) Rep68_octlink_ sediments as a monomer with sedimentation coefficient of 3.5S. (C) Rep68_octlink_ in presence of 5 mM ATP sediments in two peaks corresponding to monomer and dimer species. Protein concentration was kept constant at 25 µM in buffer containing 200 mM NaCl. Sedimentation velocity experiments were run at 40000 rpm and 20°C. Data was collected using the interference system.

To determine if the linker is critical for the oligomerization of Rep68, we replaced it with an unrelated sequence and examined its effect on oligomerization using sedimentation velocity. The only prerequisite for the substitute linker were a lack of structure and no impact on the native structures of the connected domains. We chose a sequence from the transcription factor Oct-1. This transcription factor has two DNA binding domains connected by a linker of 29 residues. The X-ray structure of this protein shows that the linker is unstructured and flexible. In addition, it has been used to connect different protein domains without affecting their properties [25], [26]. We generated a Rep68 mutant protein (Rep68_octlink_), where residues 206 to 224 were replaced with 18 residues from the Oct-1 linker and tested its ability to oligomerize. The sedimentation profile of Rep68 typically shows two populations with sedimentation coefficients of ∼3S and ∼13S (Figure 6A). We have determined that the 13S peak corresponds to a mixture of oligomeric rings (data not shown). Figure 6B shows that the replacement of the linker completely abolishes the oligomerization of the mutant protein Rep68_octlink_. We can detect formation of dimeric species only at the highest concentration tested and in the presence of ATP, (Figure 6C). These results show that replacement of the linker produces a Rep68 protein whose ability to oligomerize has been severely affected.

### Presence of the linker region induces oligomerization of the OBD domain

**Figure 7:**
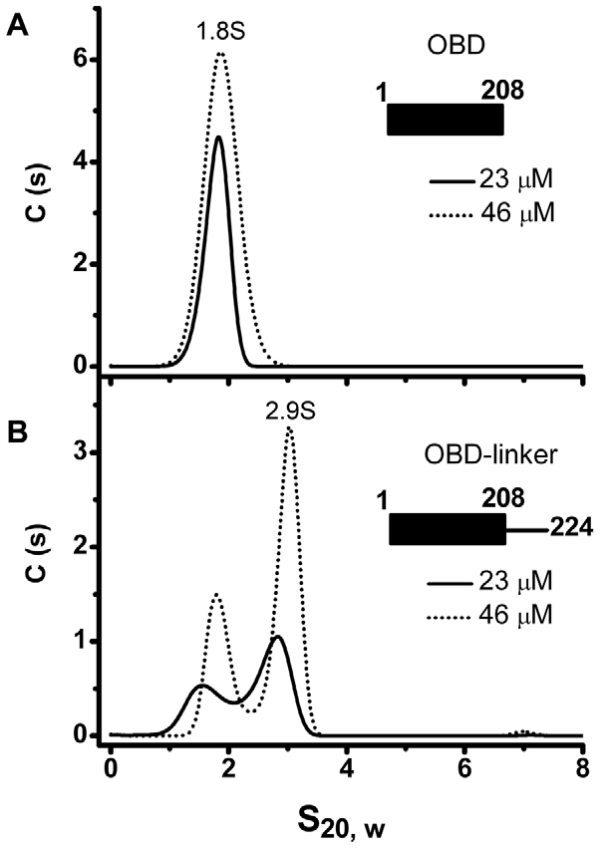
Oligomerization of the OBD domain is induced by the interdomain linker. (A) Sedimentation profile of the OBD (residues 1–208) at two different protein concentrations (23 and 46 µM). (B) Sedimentation profile of OBD-linker (residues 1–224) shows monomers and dimers sedimenting at ∼1.8S and ∼2.9S respectively. Sedimentation velocity experiments were run at 40000 rpm and 20°C. Data was collected using the interference system.

The above findings indicate that the linker region plays a central role in the oligomerization of AAV Rep proteins. To confirm that the linker region has an intrinsic property to induce oligomerization, we generated a construct that spans the OBD domain and the linker region (OBD-linker residues 1–224) and measured its ability to oligomerize. We first analyzed the OBD domain (1–208) to determine any oligomerization up to concentrations of 1 mg/ml (43 µM). Our results show that while OBD is a monomer (Figure 7A), the OBD-linker protein construct displays formation of dimers at increasing protein concentrations (Figure 7B). These results support the hypothesis that the linker region has an intrinsic property to induce oligomerization

### Linker residue Y224 is critical for oligomerization and represents a conserved feature in SF3 helicases

**Figure 8:**
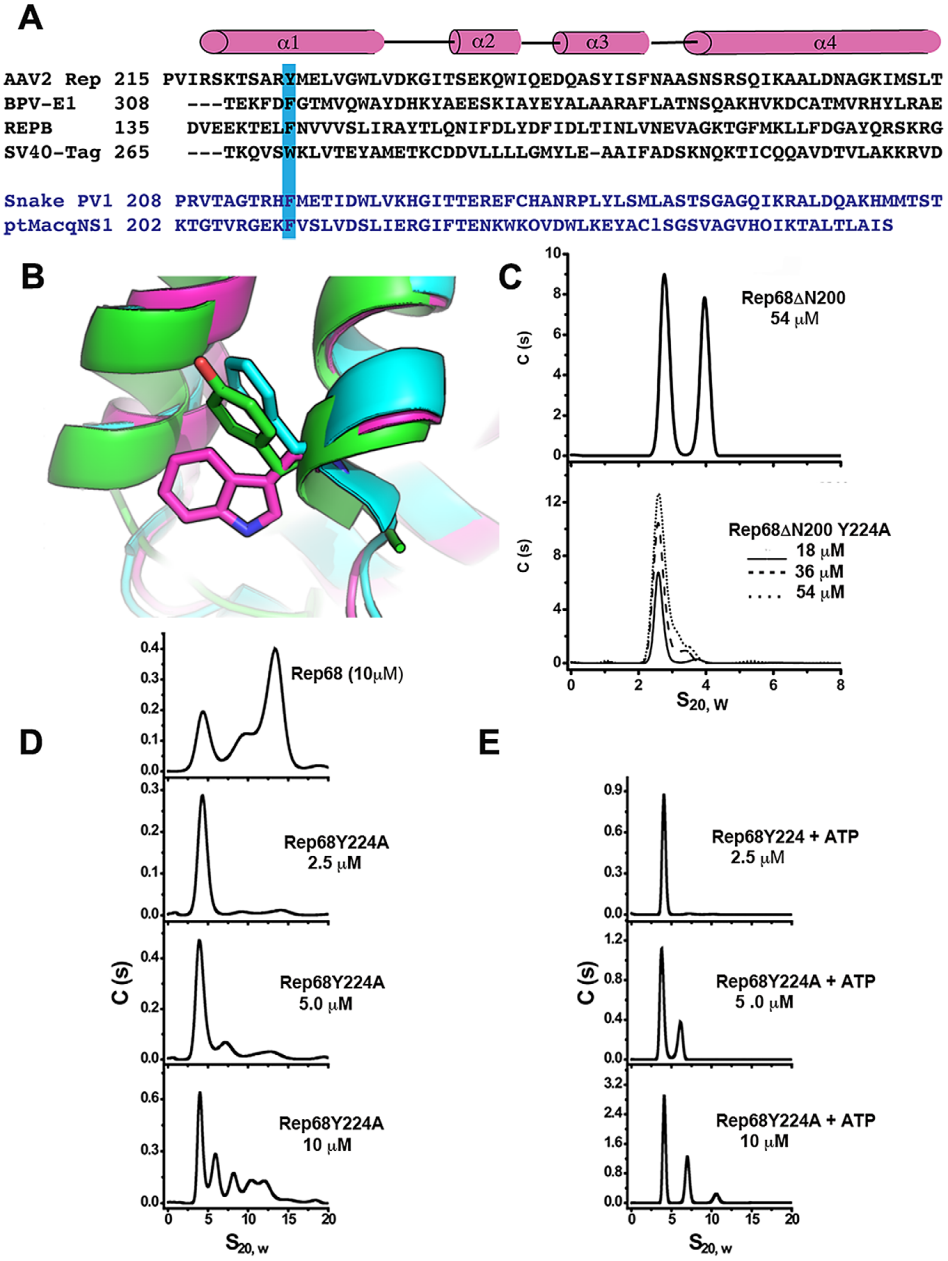
Residue Y224 is important for oligomerization. (A) Structure-based sequence alignment of four OD domains: AAV2 Rep, Adeno-Associated virus 2 Rep; BPV-E1, Bovine papillomavirus E1 protein; REPB, Plasmid pMV158 Replication initiator protein; SV40-Tag, Simian virus 40 large T antigen. Also shown in blue the putative OD domains sequences from Snake PV1, Snake parvovirus 1 and ptMacq NS1, Point-tailed Macaque parvovirus non-structural protein 1. Highlighted in blue are the conserved aromatic residues. (B) Ribbon diagram of the structural alignment showing the aromatic residues for AAV-2 Rep (green), SV40-Tag (magenta) and BPV-E1 (Blue). (C) Sedimentation profiles of Rep68ΔN200 and Rep68ΔN200Y224A constructs. (D) Rep68 protein is compared with the mutant Rep68Y224A at different concentrations (2.5 to 10 µM). (E) ATP effect on the oligomerization of Rep68Y224A mutant. Concentration of protein was varied as in figure D from 2.5 µM to 10 µM. ATP and MgCl_2_ were at 5 mM.

We generated a model of the Rep68ΔN214 construct using the X-ray structure of Rep40 (residues 225–490) and 9 residues of the linker (215–224) that were added as a helical extension to the N-terminus. The model of the α-helix was generated using Robetta [27]. Figure 8A and 8B shows the structural alignment of the OD domain of the Rep68ΔN214 model with the OD domains of PV-E1 and SV40-LTag. The alignment shows that residue Y224 superimposes with aromatic residues F313 and W270 located at the beginning of helix 1 in the OD domains of PV-E1 and SV40-LTag respectively. Analysis of the structures of both proteins reveals that these aromatic residues play a critical role in forming and stabilizing the oligomerization interface. They pack against both the N-terminal end of helix 4 of the same subunit and the C-terminus end of helix 4 of the neighboring subunit. In order to test the hypothesis that Y224 plays an equivalent role in AAV Rep proteins, we mutated it to alanine and tested its effect on the oligomerization of Rep68ΔN200. Mutation to the smaller residue alanine should have a direct effect in the oligomerization of this protein because of the significant reduction of surface exposed area. Figure 8C shows the sedimentation profile of this mutant protein showing that it completely abolishes the formation of dimers. To confirm that residue Y224 plays an important role in the oligomerization of AAV Rep proteins, we generated a Rep68Y224A mutant and compared its ability to form oligomers with respect to wild type Rep68. Analysis of the Rep68Y224A mutant reveals that at low concentration the protein is mostly found as a monomer with a sedimentation coefficient of ∼3S. At higher concentrations, we observed the appearance of multiple peaks that correspond to dimers, trimers and larger oligomers; nevertheless, the majority of the protein is present as a monomer. The presence of ATP induces a small degree of stability to the dimeric species at 5 µM and both the 5S and 11S species at 10 µM. However, the 13S complex observed with the wild type Rep68 is not formed and most of the protein is still found as a monomer (Figure 8E). These results indicate that residue Y224 is critical for the oligomerization of AAV Rep proteins.

### Residue Y224 is critical for AAV virus viability

**Figure 9:**
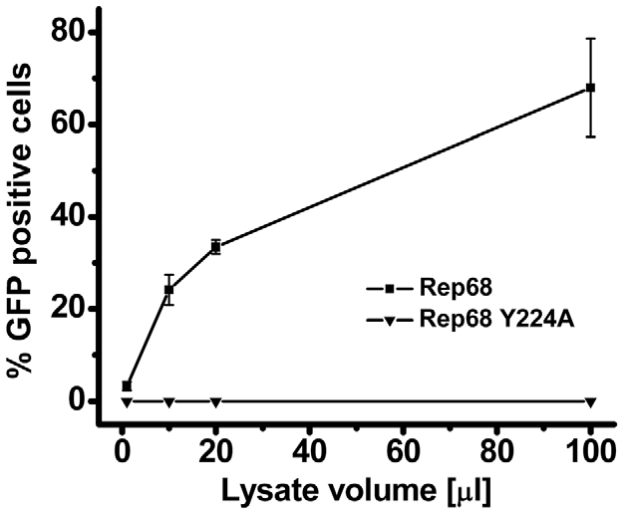
Effect of Y224 mutation on AAV-2 virus liability. Comparison of the production of rAAV2-GFP infectious particles in presence of wt (squares) or Y224A Rep (triangles). rAAV2-GFP particles were produced in 293T cells in presence of wt or Y224A Rep. Varying volumes of crude lysate (in µl, x-axis) were added to HeLa cells and the percentage GFP positive -infected- cells was determined by FACS analysis. Data from four experiments are represented as mean ± s.e.m.

To assess if the disruption of oligomerization observed with the Rep68Y224A mutant has any consequences on the AAV viral life cycle, we produced recombinant AAV2 particles expressing the GFP gene in presence of a helper virus containing the Y224A mutation in the Rep ORF. The cells were harvested and lysed, and the crude lysate (treated with an endonuclease) was used to infect Hela cells. Strikingly, the crude lysate from cells transfected with the mutant helper plasmid didn't contain any infectious rAAV2-GFP particles, as determined by FACS analysis of GFP positive cells (Figure 9). These results show that the residue Y224 of AAV Rep proteins, and the oligomeric properties it confers to these proteins, have a crucial role during the AAV life cycle.

## Discussion

In this study we report that the interdomain linker present in the larger AAV Rep68/78 proteins is an integral part of their oligomerization interface. We showed that the linker region is in fact an extension of the OD domain of AAV Rep proteins. Our results have shown that Rep40 constructs containing either a complete or half linker have the ability to oligomerize. This effect is enhanced in presence of ATP or ADP. We hypothesized that the linker region from residues 215 to 224 forms a α-helix that is connected to the first α-helix of the SF3 helicase domain. Secondary structure prediction and modeling of the linker region supports this argument (Figure 3A and 8B). Furthermore, we have identified a critical aromatic residue (Y224) located at the end of the linker region that is conserved in Rep proteins from all AAV serotypes. The bulky nature of this aromatic residue appears to be a conserved feature in SF3 helicases (Figure 8A). Structural alignment of the OD domain of a Rep40 model with an extended helical linker and those of SV40-LTag and PV-E1 shows that residue Y224 aligns with equivalent aromatic residues Trp270 and Phe313 respectively (Figure 8A, 8B). A detailed analysis of the oligomeric interface of these proteins shows that these aromatic residues have a dual role: they stabilize the hydrophobic core of the OD domain helical bundle, and are part of the oligomerization interface between neighboring subunits. Our results reveal the critical role of the OD domain in the formation of stable oligomers in SF3 helicases. The larger OD domains of SV40-Tag and PV-E1 proteins in cooperation with the AAA^+^ motor domain generate a helicase domain that forms stable hexamers. Constructs of SV40-LTag and PV-E1 without the OD domain fail to oligomerize [14], [19]. Another example that shows the fundamental role of the OD domain in oligomerization comes from the study of the evolutionary related proteins involved in rolling circle replication (RCR) of plasmids. The protein RepB from streptococcal RCR plasmid pMV158 is a hexameric protein that initiates replication of plasmid DNA and has a domain structure that resembles SF3 helicases but lacks the AAA^+^ subdomain [28]. Its N-terminal OBD domain is structurally and functionally related to the OBD from AAV Rep proteins due to the presence of the HUH motif critical for DNA nicking. Its C-terminal domain only consists of a 4 helical bundle that is similar to the OD domains of SF3 helicases and is responsible for hexamerization. Structural alignment shows that RepB has an aromatic residue (Phe143) equivalent to residue Y224 in AAV Rep68/78. We hypothesize that the role of this residue has been conserved throughout evolution to serve as a modulator of oligomerization in SF3 helicases and related RCR proteins. The smaller AAV Rep proteins Rep40/52 with truncated OD domains are missing the Y224 residue and thus are not able to sustain a stable oligomerization interface and are mostly monomeric. Consequently, the stable oligomerization of AAV Rep proteins requires the cooperative interaction of the OBD domain, the linker and the helicase domain. In this context, the OD sub-domain, and in particular the aromatic residue at the C-terminus of linker, appear to be the triggering element required for the oligomerization of AAV Rep proteins.

The critical role of residue Y224 in the overall AAV-2 viral life cycle is illustrated by the complete abolishment of production of infectious particles from AAV-2 vector constructs produced in the context of Rep carrying the Y224A mutation (Figure 9). This result prompts the question of which specific functions are affected by this mutation. We think that most of the biochemical activities of Rep68/78 will be affected due to the impairment in oligomerization. Remarkably, an earlier report by Walker et al. on the identification of residues necessary for site-specific endonuclease activity showed that a Y224 mutant was defective in AAV hairpin/DNA binding, trs endonuclease, DNA helicase and ATPase activity [29], suggesting that correct oligomerization of Rep proteins may be important in all of these functions.

In agreement with our results, a recent report has shown that the presence of the linker in an AAV5 Rep40 construct induces oligomerization in presence of DNA. However, the authors concluded that the linker effect is primarily due to its interaction with DNA [30]. As we demonstrated in this report, the oligomerization effect is an intrinsic property of the linker due to its critical role in the formation of an oligomerization interface as part of the OD domain. The presence of DNA induces further oligomerization as seen with all helicases [13]. However, it appears that the linker also plays an additional role in protein-DNA interaction that may be important during the assembly of Rep68/78 on DNA substrates such as the AAV origin of replication and AAVS1 integration site.

The use of alternative gene promoters is a common mechanism to generate protein diversity and flexibility in gene expression. At the same time it allows to obtain multiple functions from a limited number of genes, thus optimizing the size of the genome. It is clear that in the case of the Rep proteins from the AAV virus, nature has generated two sets of proteins that differ primarily in their ability to oligomerize. Rep proteins obtained from the AAV P_19_ promoter generate Rep40 and Rep52 with truncated OD domains and are thus unable to oligomerize. Both proteins play a critical role during DNA packaging into capsids; however, the mechanism of action of monomeric Rep40/52 during packaging remains elusive. Rep proteins generated from the P_5_ promoter, on the other hand, require the cooperative interaction of three different oligomeric interfaces produced by the OBD domain, the linker and the helicase domain. This feature potentially provides an additional dimension for the regulation of the diverse Rep activities when compared to the related proteins from SV40 and PV. We suggest that the cooperative interactions and the modulation of these interfaces – in particular in the presence of various specific DNA substrates – orchestrate the variety of functions performed by Rep68/Rep78 proteins and may thus represent a key to our understanding of the underlying mechanisms.

Finally, our report introduces the possibility of two distinct helicase modes for the biological functions supported by AAV Rep proteins. In the context of the large Rep proteins, a complete OD domain directs the formation of stable oligomers with a DNA unwinding mode likely to resemble that of the related viral proteins SV40-Tag and E1. The small Rep proteins, however, appear to utilize an incomplete OD domain that retains Rep40/52 in a monomeric state with formation of transitional dimeric complexes required for ATP hydrolysis. It is intriguing to speculate that this unique arrangement allows AAV to utilize two distinct motor activities with a single AAA^+^ domain. As Rep40/52 have been demonstrated to be required for genome packaging it is feasible to address the question whether this process requires a Rep40/52–mediated dimeric DNA helicase activity by a mechanism that is as yet undiscovered or whether further oligomerization is induced by interaction with capsid proteins.

## Materials and Methods

### Cloning and mutagenesis of Rep expression constructs

All mutant proteins were generated using the pHisRep68/15b plasmid, which contains the AAV2 Rep68 ORF subcloned in vector PET-15b (Novagen). Site-directed mutagenesis for mutants Y224A was generated using the QuickChange mutagenesis kit (Stratagene). Rep constructs with different linker extensions were generated by PCR with primers designed to encompass the particular protein region. Primers included restriction enzyme sites NdeI and XhoI, and the sequence of the TEV protease site. The Rep68 protein used in these studies contained a Cys to Ser mutation that prevented aggregation but was functionally identical to the wild type protein (data not shown). The Rep68_octlink_ construct was generated by substitution of residues 206 to 224 of AAV2 Rep68 with the mouse Oct-1 linker residues 328–346 (GeneBank CAA49791) using the gene synthesis services from GeneScript. The sequences of all constructs were confirmed by DNA sequencing (GeneWiz).

### Protein expression and purification

All proteins were expressed using the pET-15b vector, expressed in E. coli BL21(DE3) cells (Novagen), and purified as described before [18]. The final buffer contains (25 mM Tris-HCl [pH 8.0], 200 mM NaCl, and 2 mM TCEP). His6-PreScission Protease (PP) was expressed in BL21(DE3)-pLysS at 37°C for 3 h, in LB medium containing 1 mM IPTG. Cell pellets were lysed in Ni-Buffer A (20 mM Tris-HCl [pH 7.9 at 4°C], 500 mM NaCl, 5 mM Imidazole, 10% glycerol, 0.2% CHAPS, and 1 mM TCEP). After five 10-s cycles of sonication, the fusion protein was purified using a Ni-column – equilibrated in Ni-buffer A. Protein eluted was desalted using buffer A and a HiPrep™ 26/10 desalting column (GE Healthcare). His-PP tag was removed by PreScission protease treatment using 150 µg PP/mg His-PP-Rep68. After overnight incubation at 4°C, buffer was exchanged using the same desalting column and Ni-Buffer A. Subsequent Ni-column chromatography using the buffer B (same as buffer A but with 1 M imidazole), was performed to remove the uncleaved fusion protein, and untagged Rep68 was eluted with 30 mM imidazole. Rep68 was finally purified by gel filtration chromatography using a HiLoad Superdex 200 16/60 column (GE Healthcare) and Size Exclusion buffer. N-terminus His6-tagged WT and mutant Rep68 proteins were concentrated to 10 mg/ml, flash-frozen in liquid N_2_, and kept at −80°C until use.

### Cross-linking of Rep40

The cross-linking reactions for Rep40 and Rep68ΔN200 were made according to an adapted protocol from Packman and Perham [31]. The reaction mixture was in cross-linking buffer (25 mM HEPES, 200 mM of NaCl, pH 8.0) and protein concentration was 2 mg/ml. A 30 fold molar excess of 100 mM DMP (dimethyl pimelimidate dihydrochloride, MP Biomedicals, LLC) was added to the reaction and incubated 60 min at room temperature. The reaction was quenched by addition of 1 M Tris, pH 7.5 to a final concentration of 50 mM. The samples were analyzed in an 8% SDS-PAGE.

### AAV Infectious particles assay

Hek 293T cells were triple transfected using polyethylenimine (PEI) with an AAV2 ITR-containing plasmid including the GFP gene, a helper plasmid expressing AAV2 Rep (wt or Y224A cloned from the pHisRep68Y224A/15b) and Cap, and a third construct containing the adenovirus helper functions (pXX6, University of North Carolina Vector Core Facility). The presence of the Y224A mutation was confirmed by sequencing (Eurofins). After 72 h, the cells were harvested and lysed in 150 mM NaCl, 50 mM Tris at pH 8.5, followed by three freeze - thaw cycles. The lysate was treated for 30 minutes at 37°C with 150 units/ml of benzonase endonuclease (Sigma). HeLa cells were infected with increasing amounts of crude lysate, and the percentage of GFP-positive cells was determined three days post-infection.

### Analytical ultracentrifugation

Sedimentation velocity experiments were carried out using a Beckman Optima XL-I analytical ultracentrifuge (Beckman Coulter Inc.) equipped with a four and eight-position AN-60Ti rotor. Rep protein samples were loaded in the cells, using in all cases buffer used in the final purification step. Samples in double sector cells were centrifuged at 25,000 rpm for Rep68 proteins (Rep68 and Rep68Y224A). For Rep40 and linker constructs sedimentation was performed at 40,000 rpm. In all experiments, temperature was kept at 20°C. Sedimentation profiles were recorded using UV absorption (280 nm) and interference scanning optics. For the analysis of the results the program Sedfit was used to calculate sedimentation coefficient distribution profiles using the Lamm [21].

### Structure analysis and modeling

Structures of AAV-2 Rep40 (1S9H), Bovine papillomavirus E1 protein (2GXA), Simian virus 40 T large antigen (1SVO) and plasmid pMV158 RepB (3DKY) were analyzed using the programs COOT [32], PYMOL [33] and CHIMERA [34]. Structural alignment was done using the DALI server [35]. Secondary structure prediction was performed using PredictProtein [36]. Modeling of the linker region was done using ROBETTA [27].

## References

[1] Green MR, Roeder RG (1980). Transcripts of the adeno associated virus genome: mapping of the major RNAs.. J Virol.

[2] Lusby EW, Berns KI (1982). Mapping of the 5′ termini of two adeno-associated virus 2 RNAs in the left half of the genome.. J Virol.

[3] Srivastava A, Lusby EW, Berns KI (1983). Nucleotide sequence and organization of the adeno-associated virus 2 genome.. J Virol.

[4] Yoon M, Smith DH, Ward P, Medrano FJ, Aggarwal AK (2001). Amino-terminal domain exchange redirects origin-specific interactions of adeno-associated virus rep78 in vitro.. J Virol.

[5] Cathomen T, Collete D, Weitzman MD (2000). A chimeric protein containing the N-terminus domain of the adeno-associated virus Rep protein recognizes its target site in an in vivo assay.. J Virol.

[6] Davis MD, Wu J, Owens RA (2000). Mutational analysis of adeno-associated virus type 2 Rep68 protein endonuclease activity on partially single-stranded substrates.. J Virol.

[7] Im DS, Muzyczka N (1990). The AAV origin binding protein Rep68 is an ATP-dependent site-specific endonuclease with DNA helicase activity.. Cell.

[8] Chiorini JA, Wiener SM, Owens RA, Kyostio SR, Kotin RM (1994). Sequence requirements for stable binding and function of Rep68 on the adeno-associated virus type 2 inverted terminal repeats.. J Virol.

[9] Kyostio SR, Wonderling RS, Owens RA (1995). Negative regulation of the adeno-associated virus (AAV) P5 promoter involves both the P5 rep binding site and the consensus ATP-binding motif of the AAV Rep68 protein.. J Virol.

[10] Hickman AB, Ronning DR, Kotin RM, Dyda F (2002). Structural unity among viral origin binding proteins: crystal structure of the nuclease domain of adeno-associated virus Rep.. Mol Cell.

[11] Luo X, Sanford DG, Bullock PA, Bachovchin W (1996). Structure of the origin specific DNA binding domain from simian virus 40 T-antigen.. J Virol.

[12] Enemark EJ, Chen G, Vaughn DE, Stenlund A, Joshua- Tor L (2000). Crystal structure of the DNA binding domain of the replication initiation protein E1 from papillomavirus.. Mol Cell.

[13] Singleton MR, Dillingham MS, Wigley DB (2007). Structure and mechanism of helicases and nucleic acid translocases.. Annu Rev Biochem.

[14] Sedman J, Stenlund A (1998). The papillomavirus E1 protein forms a DNA-dependent hexameric complex with ATPase and DNA helicase activities.. J Virol.

[15] Enermark E, Joshua-Tor L (2006). Mechanism of DNA translocation in a replicative hexameric helicase.. Nature.

[16] Ruiz-Maso JA, Lopez-Zumel C, Menendez M, Espinosa M, del Solar G (2004). Structural features of the initiator of replication protein RepB encoded by the promiscuous plasmid pMV158.. Biochim Biophys Acta.

[17] Smith RH, Kotin RM (1998). The Rep52 gene product of adeno-associated virus is a DNA helicase with 3′-to-5′ polarity.. J Virol.

[18] Mansilla-Soto J, Yoon-Robarts M, Rice WJ, Arya S, Escalante CR (2009). DNA structure modulates the oligomerization properties of the AAV initiator protein Rep68.. PLoS Pathog.

[19] Dawei L, Zhao R, Lilyestrom W, Gai D, Zhang R (2003). Structure of the replicative helicase of the oncoprotein SV40 large tumour antigen.. Nature.

[20] Dignam SS, Collaco RF, Bieszczad J, Needham P, Trempe JP (2007). Coupled ATP and DNA binding of adeno-associated virus Rep40 helicase.. Biochemistry.

[21] Schuck P (2000). Size-distribution analysis of macromolecules by sedimentation velocity ultracentrifugation and lamm equation modeling.. Biophys J.

[22] Kar SR, Kingsbury JS, Lewis MS, Laue TM, Schuck P (2000). Analysis of transport experiments using pseudo-absorbance data.. Anal Biochem.

[23] Correia JJ, Stafford WF (2009). Extracting Equilibrium Constants from Kinetically Limited Reacting Systems.. In: Methods Enzymol. Academic Press..

[24] James JA, Aggarwal AK, Linden RM, Escalante CR (2004). Structure of adeno-associated virus type 2 Rep40-ADP complex: insight into nucleotide recognition and catalysis by superfamily 3 helicases.. Proc Natl Acad Sci U S A.

[25] Klemm JD, Rould MA, Aurora R, Herr W, Pabo CO (1994). Crystal Structure of the Oct-1 POU domain bound to an octamer site: DNA recognition with tethered DNA-binding modules.. Cell.

[26] Panne D M, T Harrison, S.C (2004). Crystal Structure of ATF-2/c-Jun and IRF-3 bound to the interferon-B enhancer.. EMBO J.

[27] Kim DE, Chivian D, Baker D (2004). Protein Structure prediction and analysis using the Robetta server.. Nucleic Acids Res.

[28] Boer DR, Ruiz-Maso JA, Lopez-Blanco AG, Vives-Llacer M, Chacon P (2009). Plasmid replication initiator RepB forms a hexamer reminescent of ring helicases and has mobile nuclease domains.. EMBO J.

[29] Walker SL, Wonderling RS, Owens RA (1997). Mutational Analysis of the Adeno-Associated Virus Rep68 Protein: Identification of Critical Residues Necesssary for Site-Specific Endonuclease Activity.. J Virol.

[30] Maggin JE, James JA, Chappie JS, Dyda F, Hickman AB (2012). The amino Acid Linker between the Endonuclease and Helicase Domains of Adeno-Associated virus Type 5 Rep Plays a Critical Role in DNA-Dependent Oligomerization.. J Virol.

[31] Packman LC, Perham RN (1982). Quaternary structure of the pyruvate dehydrogenase multienzyme complex of Bacillus sterearothermophilus studied by a new reversible cross-linking procedure with bis(imidoesters).. Biochemistry.

[32] Emsley P, Lohkamp B, Scoot WG, Cowtan K (2010). Features and development of Coot.. Acta Crystallogr D Biol Crystallogr.

[33] Schrodinger LLC (2010). PyMOL The PyMOL Molecular Graphics System. Version 1.3.. Schrodinger,LLC.

[34] Pettersen EF, Goddard TD, Huang CC, Couch GS, Greenblatt DM (2004). UCSF Chimera - A Visualization System for Exploratory Research and Analysis.. J Comput Chem.

[35] Holm L, Rosenström P (2010). Dali server: conservation mapping in 3D.. Nucleic Acids Res.

[36] Rost B, Yachdav G, Liu J (1997). The PredictProtein Server.. Nucleic Acids Res.

